# Unraveling the Activation Mechanism of the Potato Tuber ADP-Glucose Pyrophosphorylase

**DOI:** 10.1371/journal.pone.0066824

**Published:** 2013-06-24

**Authors:** Carlos M. Figueroa, Misty L. Kuhn, Christine A. Falaschetti, Ligin Solamen, Kenneth W. Olsen, Miguel A. Ballicora, Alberto A. Iglesias

**Affiliations:** 1 Instituto de Agrobiotecnología del Litoral, Universidad Nacional del Litoral and Consejo Nacional de Investigaciones Científicas y Técnicas, Santa Fe, Santa Fe, Argentina; 2 Department of Chemistry and Biochemistry, Loyola University Chicago, Chicago, Illinois, United States of America; Consiglio Nazionale delle Ricerche, Italy

## Abstract

ADP-glucose pyrophosphorylase regulates the synthesis of glycogen in bacteria and of starch in plants. The enzyme from plants is mainly activated by 3-phosphoglycerate and is a heterotetramer comprising two small and two large subunits. Here, we found that two highly conserved residues are critical for triggering the activation of the potato tuber ADP-glucose pyrophosphorylase, as shown by site-directed mutagenesis. Mutations in the small subunit, which bears the catalytic function in this potato tuber form, had a more dramatic effect on disrupting the allosteric activation than those introduced in the large subunit, which is mainly modulatory. Our results strongly agree with a model where the modified residues are located in loops responsible for triggering the allosteric activation signal for this enzyme, and the sensitivity to this activation correlates with the dynamics of these loops. In addition, previous biochemical data indicates that the triggering mechanism is widespread in the enzyme family, even though the activator and the quaternary structure are not conserved.

## Introduction

Starch and sucrose are the major photosynthetic products in most plants [1]. During light hours, fixed carbon is in part used for starch synthesis within chloroplasts. In the night, starch is remobilized to cope with the plant’s demands. Starch synthesized in leaves is considered a transitory molecule, whereas in sink tissues (such as roots, fruits, and tubers) it constitutes a long-term storage of carbon and energy. Starch is synthesized in both source and sink tissues by a series of reactions, including: i) synthesis of ADP-glucose (ADP-Glc); ii) transfer of the Glc moiety to a preformed α-1,4-polyglucan; and iii) introduction of α-1,6 branching points into the α-1,4-polyglucan. Regulation of starch synthesis is mainly exerted at the level of the first reaction, which forms the sugar-nucleotide [2], [3]. This is critical for a correct coordination of metabolic fluxes of carbon in the different plant cells [1].

ADP-Glc pyrophosphorylase (ADP-Glc PPase, EC 2.7.7.27) catalyzes the first committed step of glycogen and starch synthesis in bacteria and plants, respectively. This enzyme converts ATP and Glc-1P into ADP-Glc and inorganic pyrophosphate (PPi) in the presence of a divalent cation (Mg^2+^) [2], [3]. Most ADP-Glc PPases from bacteria (including cyanobacteria) are homotetramers, whereas the enzymes from green algae and plants are composed of two small (S) and two large (L) subunits. These subunits share a common ancestor, and their roles have co-evolved via a subfunctionalization mechanism [4]. Depending upon the organism and the tissue, one or both subunits can play catalytic and modulatory roles [4]–[6]. In the potato tuber enzyme, the first to have its subunit roles characterized, the S subunit is mostly catalytic, with a deficient regulation. The L subunit, however, is non-catalytic and plays a modulatory role by interacting with the S subunit and increasing its affinity for the activator [7].

ADP-Glc PPase exemplifies how a metabolic pathway can be strongly controlled by an allosteric mechanism. ADP-Glc PPases are regulated by intermediaries of the major carbon assimilation pathway, and this regulation varies among different organisms [2], [3]. For instance, the enzyme from *Escherichia coli* is activated by fructose-1,6-bisphosphate (Fru-1,6-bisP) and inhibited by AMP, while that from *Agrobacterium tumefaciens* is activated by fructose-6-phosphate and pyruvate and inhibited by AMP and ADP. On the other hand, ADP-Glc PPases from oxygenic photosynthetic organisms (cyanobacteria, green algae, and plants) are mainly regulated by 3-phosphoglycerate (3-PGA, activator) and inorganic orthophosphate (Pi, inhibitor) [2], [3].

Chemical modification of spinach leaf ADP-Glc PPase [8], [9] and site-directed mutagenesis of the *Anabaena* sp. PCC 7120 [10], [11] and potato tuber [12] enzymes showed that Lys residues important for 3-PGA and Pi binding are located in the C-terminal domain, whereas substrates bind to the N-terminal domain. There is strong evidence that activators and inhibitors of the ADP-Glc PPase family bind at the interface of the C- and N-terminal domains. For instance, the crystal structure of the S subunit from potato tuber (S_4_) has two sulfates (analogs of Pi, the inhibitor) bound at this interface, which interact with the Lys residue responsible for 3-PGA and Pi binding [13]. Residues involved in Fru-1,6-bisP binding were found in the N-terminal domain of the *E. coli* enzyme [14]. Similarly, residues important for fructose-6-phosphate and pyruvate binding in the *A. tumefaciens* enzyme were identified in the N-terminal region [15]. These residues are also located at the interface between the C- and N-terminal domains. Interestingly, hybrid proteins composed of the *E. coli* N-terminal and the *A. tumefaciens* C-terminal domains (and vice versa) revealed that interaction of both domains plays a critical role for determining the specificity for the activator [16].

The crystal structures of the homotetrameric (S_4_) potato tuber ADP-Glc PPase [13] and of the *A. tumefaciens* enzyme [17] were reported within the last decade. In the *E. coli* ADP-Glc PPase, a critical region for the allosteric activation involving three nearby loops was identified by modeling and biochemical characterization of a random insertion of five amino acids into the enzyme (Ins8) [18]. Thereafter, highly conserved amino acids Gln74 and Trp113, located in two of those loops, were mutated in the *E. coli* enzyme [19]. Mutant enzymes Q74A and W113A mimicked the properties of the Ins8 random mutant characterized by Ballicora et al. [18]. They were insensitive to Fru-1,6-bisP activation, even when the activator was bound to the enzyme. Based upon these results, we proposed a location for the allosteric activator and a mechanism by which the regulatory signal propagates to trigger a more active and closed conformation [19].

The question remained as to whether this type of mechanism is specific for Fru-1,6-bisP or whether any orthologous ADP-Glc PPase activated by another allosteric regulator can exhibit a similar activation mechanism. To investigate this question, we characterized specific loops involved in the allosteric activation of ADP-Glc PPases from the oxygenic photosynthetic organisms *Anabaena* and *Solanum tuberosum* (potato, tuber tissue). The side-by-side characterization of these two enzymes also allowed us to compare how activation by 3-PGA is exerted when the enzyme is either a homotetramer or heterotetramer (both catalytic and regulatory subunits).

## Materials and Methods

### Materials

α-D-[U-^14^C]Glc-1P was purchased from GE Healthcare (Piscataway, NJ, USA). Substrates, effectors and enzymes used for kinetic determinations were purchased from Sigma (Saint Louis, MO, USA). Phusion DNA polymerase and restriction enzymes were purchased from New England Biolabs (Ipswich, MA, USA). StrataClone Blunt PCR cloning kit was purchased from Agilent Technologies (Santa Clara, CA, USA). All the other reagents were of the highest quality available. *Escherichia coli* glycogen synthase used in *Assay B* was expressed and purified as previously described [20].

### DNA Methods

#### Mutants of the*Anabaena* ADP-Glc PPase

Mutations were introduced using the QuikChange kit (Agilent Technologies) and the plasmid pAnaE3a [21], which contains the sequence coding for the *Anabaena* enzyme. PCR conditions were: initial denaturation of 1 min at 95°C, and 16 cycles of 1 min at 95°C, 1 min at 55°C, and 18 min at 72°C. Finally, PCR products were treated with *Dpn*I to remove the template.

#### Mutants of the potato tuber ADP-Glc PPase

Site-directed mutagenesis was performed by PCR overlap extension [22] using Phusion DNA polymerase. Plasmids used as templates for obtaining the different mutants were pML10 [7] and pMON17336 [23], which contain the sequences coding for the small (*Stu*S) and large (*Stu*L) subunits of the potato tuber enzyme. PCR conditions were: initial denaturation of 30 s at 98°C; 30 cycles of 5 s at 98°C, 20 s at 50°C, and 1 min at 72°C; final extension of 5 min at 72°C. The PCR products were purified after agarose gel electrophoresis and inserted into the pSC-B vector using the StrataClone Bunt PCR kit. After silent mutations were introduced in both genes to remove *Nde*I sites, punctual mutations were introduced to obtain the desired mutants. The genes coding for *Stu*S, *Stu*S_Q75A_, and *Stu*S_W116A_ were subcloned into the pMAB5 vector using *Nde*I and *Sac*I sites; whereas the genes encoding *Stu*L, *Stu*L_Q86A_, and *Stu*L_W128A_ were subcloned into the pMAB6 vector between *Nde*I and *Sac*I sites [24].

Oligonucleotides (Table S1) were synthesized by Integrated DNA Technologies (Coralville, IA, USA). All mutations were confirmed by automated DNA sequencing at the Cancer Research Center, University of Chicago (Chicago, IL, USA).

### Enzyme Expression and Purification

The recombinant enzymes were expressed in *E. coli* AC70R1-504 cells, which are defective in endogenous ADP-Glc PPase activity [23]. To obtain the different heterotetrameric enzymes, cells were transformed with the plasmids harboring the wild type or mutant genes. Transformed cells were grown in 1 l of LB medium supplemented with the appropriate antibiotic (50 µg/ml kanamycin for pMAB5, 70 µg/ml spectinomycin for pMAB6, and 100 µg/ml ampicillin for pAnaE3a) at 30°C and 250 rpm until the OD_600 nm_ reached ∼1.2. Cells were induced with 0.4 mM isopropyl β-D-1-thiogalactopyranoside and 5 µg/ml nalidixic acid (when necessary) at 20°C and 250 rpm for 16 h. After induction, cells were harvested by centrifuging 10 min at 4°C and 5000×*g*.

Recombinant proteins were purified using the following method. The cell pastes were resuspended in *Buffer A* [50 mM HEPES pH 8.0, 5 mM MgCl_2_, 0.1 mM EDTA, 10% (w/v) sucrose] and disrupted by sonication. The resulting suspensions were clarified by centrifuging 20 min at 4°C and 20,000×*g* and the crude extracts were loaded onto a 10-ml DEAE-Sepharose column (GE Healthcare) previously equilibrated with *Buffer A*. Elution of the proteins was accomplished with a linear gradient of NaCl (20 column volumes, 0 to 0.5 M). Fractions containing ADP-Glc PPase activity were pooled and precipitated with ammonium sulfate at 70% saturation. The suspensions were centrifuged and the proteins were resuspended in *Buffer H* (*Buffer A* +1 M ammonium sulfate) and loaded onto two 1-ml Resource PHE columns (GE Healthcare) in tandem previously equilibrated with *Buffer H*. The elution was conducted with a linear gradient of ammonium sulfate (50 column volumes, 1 to 0 M). Fractions containing ADP-Glc PPase activity were pooled, desalted and concentrated using an Amicon Ultra-4 30 K unit (Millipore, Billerica, MA, USA) and stored at −80°C. Under these conditions, the enzymes remained fully active for at least 3 months.

### Enzyme Assays

#### Assay A. Colorimetric method

Activity of the *Anabaena* enzymes was measured using the highly sensitive, colorimetric method developed by Fusari et al. [25]. Assays were performed in 100 mM MOPS pH 7.5, 5 mM MgCl_2_, 0.5 mM Glc-1P, 1 mM ATP, 0.5 U/ml inorganic pyrophosphatase, 0.2 mg/ml BSA, and enzyme in an appropriate dilution in a total volume of 50 µl. When necessary, 2 mM 3-PGA was added. Pi production after hydrolysis of PPi by inorganic pyrophosphatase was measured as previously described [25].

#### Assay B. Radioactive method

Activity of the potato tuber enzymes was determined using the method developed by Yep et al. [20]. The reaction mixture contained 50 mM HEPPS pH 8.0, 7 mM MgCl_2_, 4 mM DTT, 0.5 mM ∼1000 dpm/nmol [^14^C]Glc-1P, 1.5 mM ATP, 0.5 U/ml inorganic pyrophosphatase, 0.2 mg/ml BSA, and enzyme in an appropriate dilution in a total volume of 200 µl. When necessary, 5 mM 3-PGA was added. The production of [^14^C]ADP-Glc was measured by coupling the reaction catalyzed by the *E. coli* glycogen synthase, as previously described [20].

To obtain saturation curves of a given substrate or effector, its concentration was varied, while the other conditions remained constant. One unit of enzyme activity is defined as the amount of enzyme producing 1 µmol of [^14^C]ADP-Glc or PPi in 1 min at 37°C under the specified conditions.

### Kinetic Characterization

To determine the kinetic parameters of the recombinant enzymes, variable concentrations of substrates or effectors were added to the reaction assay media. Activity data was plotted against the variable substrate or effector concentration using the program Origin 7.0 (OriginLab Corporation) and fitted to a modified Hill equation: *v* = *v*_0_+(*V*–*v*_0_)*C*^n^* ^H^/(*k^n^* ^H^+C*^n^* ^H^), where *v* is the initial velocity, *v*_0_ is the velocity in absence of the substrate or effector being analyzed, *V* is the maximal velocity (*V*_max_), activation or inhibition, C is the concentration of substrate or effector under study, *k* is the concentration of substrate or effector producing half of the maximal velocity (*S*_0.5_), activation (*A*_0.5_) or inhibition (*I*_0.5_), and *n*_H_ is the Hill coefficient. Standard deviations were calculated by the fitting software. Kinetic experiments were performed at least twice with similar results.

### Protein Methods

Protein electrophoresis under denaturing conditions (SDS-PAGE) was performed in a 10% polyacrylamide gel as described by Laemmli [26]. The BenchMark protein ladder (Invitrogen, Grand Island, NY, USA) was used, and gels were stained with GelCode Blue Stain Reagent (Thermo Fisher Scientific, Rockford, IL, USA). Protein concentration after purification was determined by UV absorbance at 280 nm using a NanoDrop ND-1000 spectrophotometer (Thermo Fisher Scientific) and an extinction coefficient of 1 ml cm^−1^ mg^−1^.

### Computational Methods

Using the crystal structure of the small subunit of potato tuber ADP-Glc PPase (1YP3) [13] as a template, a model of the large subunit (*Stu*L) was generated using Modeller [27]. The same program was used to generate the missing loop (residues 90 to 98) in the small subunit (*Stu*S). No attempt was made to generate a structure for the residues missing from the N-termini of the subunits. ATP and Mg^2+^ were placed into these models in the positions found in the X-ray structure of the small subunit. In addition to the native subunits, four mutant structures were generated using VMD [28]: *Stu*S_Q75A_, *Stu*L_Q86A_, *Stu*S_W116A_, and *Stu*L_W128A_. Each simulation box, containing one subunit, ATP, Mg^2+^, a TIP3 water box extending at least 10 Å beyond the protein in all directions, and 0.1 M NaCl adjusted to neutralize the charge in the water box, was assembled using the molecular graphics program VMD [28]. The simulation box was then brought to equilibrium using the molecular dynamics program NAMD [29]. The equilibration procedure involved energy minimization with and without restraints on the protein coordinates (3000 steps each), slow heating from 10 to 310 K (30,000 steps), and then pressure and temperature equilibration using a Langevin piston (10,000 steps). Finally, unrestrained dynamics for 100,000 steps was done before data was acquired. Periodic boundary conditions were used. The cutoffs for non-bonding (van der Waals and electrostatic) interactions were 12 Å. The switch distance was 10 Å, and 1.0 1–4 scaling factor was used. All calculations were done using CHARMM 27 parameters [30]. Molecular dynamic simulations (10 ns) were created using NAMD [29] for both the wild type and mutants of each subunit. The diagrams were generated using the VMD molecular graphics program. Root mean squared fluctuations (RMSF) were computed using VMD [28] and the difference in RMSF between the mutant and the native structures were calculated for each residue. The loops missing in the X-ray structures were not included in these calculations since these areas of the structures were very highly flexible.

## Results and Discussion

**Figure 1:**
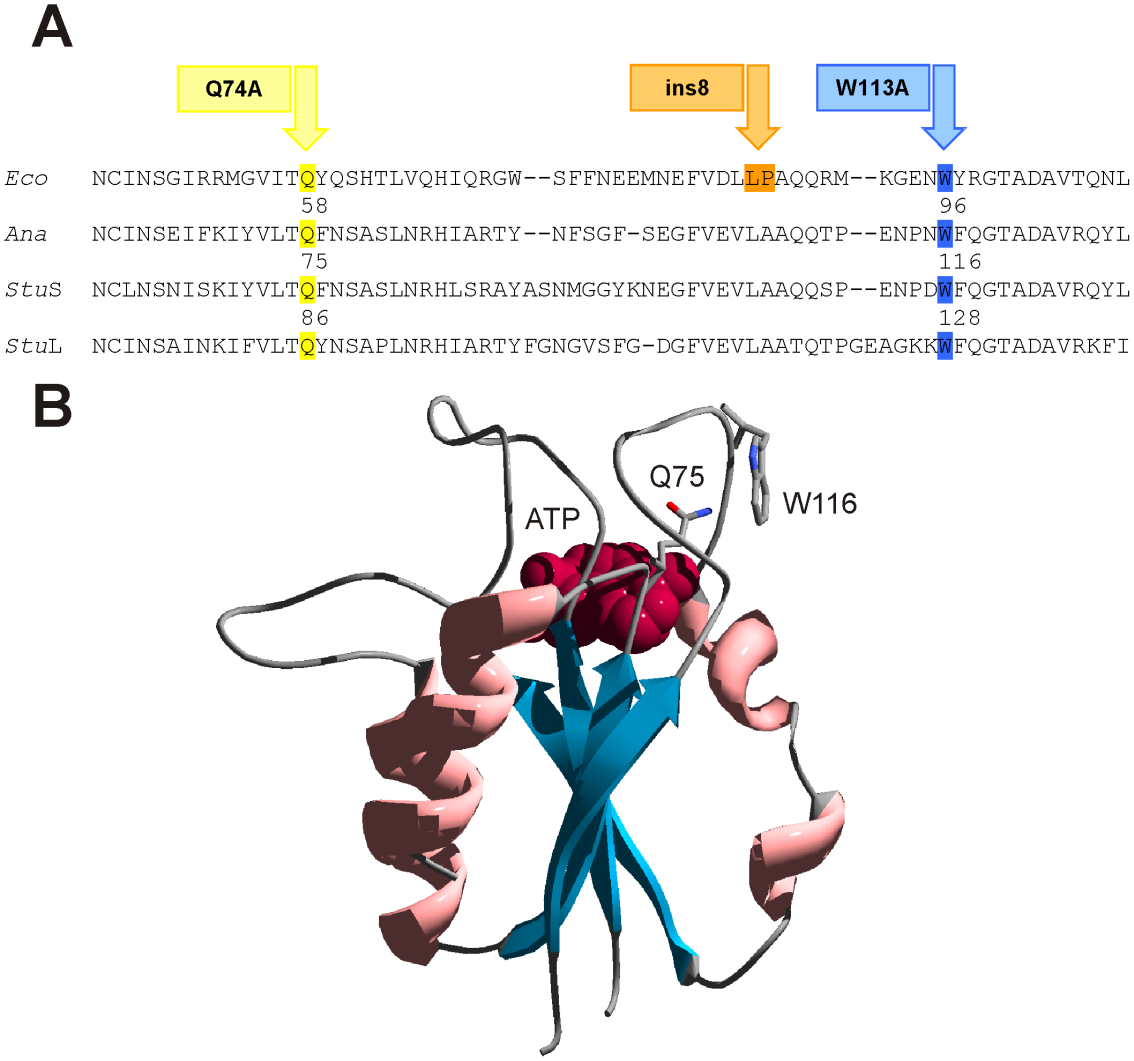
Identification of amino acids important for 3-PGA activation. **A.** Sequence alignment of ADP-Glc PPases from *Escherichia coli* (*Eco*), *Anabaena* PCC 7120 (*Ana*), and potato tuber (*Stu*S: small subunit, *Stu*L: large subunit). Mutants of the *E. coli* enzyme that were insensible to Fru-1,6-bisP activation [19] are pointed with arrows. Residues that might be involved in 3-PGA activation of ADP-Glc PPases from *Anabaena* and potato tuber are highlighted in yellow (Gln) and blue (Trp). **B.** Insight into the three-dimensional structure of the N-terminal domain (residues 20 to 144) from *Stu*S. Residues Q75 and W116 are shown as “sticks” and colored by atom type. ATP is shown in red as van der Waals radii. Loops are colored in grey, α-helices in pink, and β-helices in blue.

**Figure 2:**
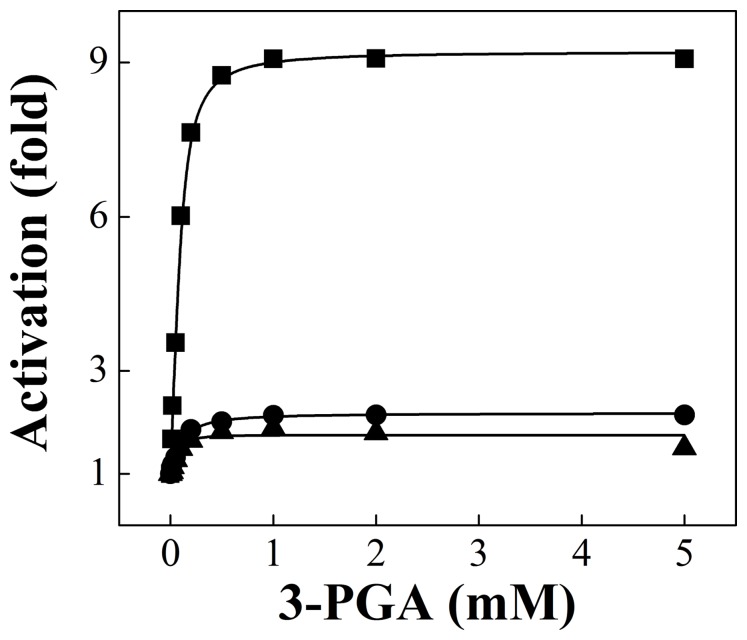
Saturation curves of 3-PGA for the wild type*Anabaena* ADP-Glc PPase (▪) and mutants *Ana*_Q58A_ (•) and *Ana*_W96A_ (▴). Reactions were performed using *Assay A*, as stated under “Materials and Methods”.

The primary sequence of the *E. coli* ADP-Glc PPase shares 31.3%, 31.8%, and 24.7% identity with those from *Anabaena* sp. PCC 7120, *Stu*S, and *Stu*L, respectively. Despite these values are relatively low, it has been demonstrated that the secondary structure and the key residues for activity and binding of substrates and/or regulatory molecules are well conserved throughout the ADP-Glc PPase family [2], [3]. The bacterial enzymes have an identical fold as it could be seen from the comparison between the X-ray structures of the *A. tumefaciens* enzyme and *Stu*S [13], [17]. The *E. coli* and the *A. tumefaciens* enzyme are 55.2% identical (71.7% similar) and chimeric constructions between them demonstrated their structural compatibility. In addition, construction of chimeras between the *Anabaena* enzyme and *Stu*S ensured they shared a common structure [31]. We recently showed that site-directed mutagenesis of residues Gln74 and Trp113 abolished Fru-1,6-bisP activation of the *E. coli* enzyme [19]. These residues are highly conserved between enzymes from different sources. Figure 1A shows the homologous residues positioned in the homotetrameric enzyme from *Anabaena* sp. PCC 7120 and from potato tuber (either in the S or L subunit). These residues are located in loops adjacent to the ATP binding site (Figure 1B). The *Anabaena* and potato tuber enzymes were selected to explore whether those residues play similar roles when the allosteric activator is the three carbon metabolite 3-PGA rather than the hexose-bisP. As a first approach, we constructed and characterized mutants of the *Anabaena* enzyme (*Ana*_WT_), specifically the Q58A (*Ana*_Q58A_) and W96A (*Ana*_W96A_) mutant proteins. Interestingly, activation by 3-PGA of *Ana*_Q58A_ and *Ana*_W96A_ was significantly lower than that observed for the wild type enzyme (Figure 2). Although the mutant enzymes were less sensitive to activation, their *A*_0.5_ values for 3-PGA were similar to that of the wild type enzyme (Table 1). Despite the difference in activation-fold by 3-PGA, the kinetic parameters for substrates of both mutant enzymes were similar to those exhibited by *Ana*_WT_ (data not shown). These results suggest that the loops of the *Anabaena* ADP-Glc PPase containing the mutated residues play a role in the activation by 3-PGA in a similar fashion to the homologous domains in the Fru-1,6-bisP regulated *E. coli* enzyme [19]. In contrast to the latter, activation of the *Anabaena* mutant enzymes is not abolished but rather significantly reduced.

**Table 1:** Kinetic parameters obtained for 3-PGA with the*Anabaena* and potato tuber ADP-Glc PPases and their mutants.

Enzyme	*A*_0.5_ (mM)	*n*_H_	*V*_max_ (U/mg)
*Ana*_WT_	0.076±0.004	1.4	24.6
*Ana*_Q58A_	0.095±0.008	1.2	0.31
*Ana*_W96A_	0.083±0.008	1.4	0.19
*Stu*S/*Stu*L	0.054±0.004	0.9	18.31
*Stu*S_Q75A_/*Stu*L	0.37±0.04	0.9	0.12
*Stu*S/*Stu*L_Q86A_	0.45±0.02	1.0	5.93
*Stu*S_W116A_/*Stu*L	0.20±0.03	1.0	3.39
*Stu*S/*Stu*L_W128A_	0.8±0.1	1.3	4.18
*Stu*S_W116A_/*Stu*L_W128A_	0.41±0.06	0.9	4.24

Reactions for the *Anabaena* and potato tuber enzymes were performed using *Assay A* and *B*, respectively, as stated under “Materials and Methods”.

As previously mentioned, residues responsible for activation of the bacterial enzyme are located at both the N- and C-terminal domains [10], [14]–[16]. Similarly, residues implicated in the activation of plant enzymes (maize [32] and potato tuber [12]) have been identified in both domains in the protein. Therefore, it seems feasible that 3-PGA could trigger a similar activation mechanism. Results obtained with the homotetrameric enzymes from *E. coli* [19] and *Anabaena* (this study) strongly suggest that the mutated residues are important for triggering activation upon binding of the activator and for stabilizing the active conformation of the enzyme. The potato tuber enzyme is composed of two catalytic (S) and two modulatory (L) subunits, constituting an excellent model to further explore the activating mechanism in a more complex system. In particular, alternating mutations introduced in each subunit allowed us to identify and study inter-subunit effects that cannot be evaluated in the homotetrameric bacterial enzymes.

To characterize the activation mechanism in the potato tuber ADP-Glc PPase, we constructed different single mutants either in the S (*Stu*S_Q75A_/*Stu*L and *Stu*S_W116A_/*Stu*L) or L (*Stu*S/*Stu*L_Q86A_ and *Stu*S/*Stu*L_W128A_) subunit, as well as the respective double mutant enzymes (*Stu*S_Q75A_/*Stu*L_Q86A_ and *Stu*S_W116A_/*Stu*L_W128A_). The heterotetrameric wild type enzyme (*Stu*S/*Stu*L) and mutants *Stu*S_W116A_/*Stu*L, *Stu*S/*Stu*L_W128A_, and *Stu*S_W116A_/*Stu*L_W128A_ were expressed and purified to a level of 80% or higher (data not shown). Activity of the mutants *Stu*S_Q75A_/*Stu*L and *Stu*S/*Stu*L_Q86A_ in crude extracts was extremely low, and not enough protein for a complete kinetic analysis after purification was recovered, though we were able to analyze activator and inhibitor kinetics. On the other hand, we could not detect activity in crude extracts from cells transformed to express the double mutant *Stu*S_Q75A_/*Stu*L_Q86A_. We hypothesize that mutation of the Gln residue in both subunits destabilizes the structure of the enzyme. Thus, it was possible to express enzymes mutated either in the S or L subunit but not when the protein had both changes simultaneously.

**Figure 3:**
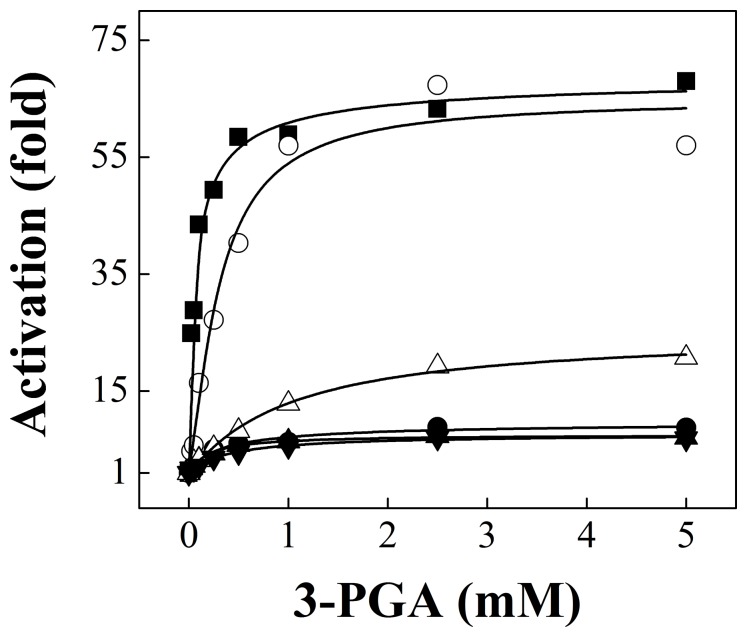
Saturation curves of 3-PGA for the wild type potato tuber ADP-Glc PPase (▪) and mutants *Stu*S_Q75A_/*Stu*L (•), *Stu*S/*Stu*L_Q86A_ (○), *Stu*S_W116A_/*Stu*L (▴), *Stu*S/*Stu*L_W128A_ (▵), and *Stu*S_W116A_/*Stu*L_W128A_ (▾). Reactions were performed using *Assay B*, as stated under “Materials and Methods”.

Saturation kinetics for 3-PGA for the wild type and mutant forms of the potato tuber ADP-Glc PPase are illustrated in Figure 3. As a general trend, all heterotetrameric mutants displayed higher *A*_0.5_ values for 3-PGA than the wild type enzyme (Table 1). When the extent of activation was analyzed, higher effects were observed when mutations were introduced in the S subunit rather than the L subunit (Table 1 and Figure 3). For instance, the behavior toward 3-PGA activation of *Stu*S_Q75A_/*Stu*L and *Stu*S_W116A_/*Stu*L was equivalent to that exhibited by the *Anabaena* mutant enzymes (compare data in Figure 3 with Figure 2), whereas *Stu*S/*Stu*L_Q86A_ was activated to a similar degree as the wild type enzyme (Figure 3 and Table 1). Comparatively, the double mutant *Stu*S_W116A_/*Stu*L_W128A_ behaved as those mutated only in the S subunit (Table 1 and Figure 3).

Details of the kinetic properties of the mutant forms of the potato tuber ADP-Glc PPase are in Table 2. As shown, the Trp mutant enzymes displayed a variety of differences in *S*_0.5_ values for ATP, Glc-1P, and Mg^2+^, relative to those of the wild type enzyme. In the absence of activator, all mutant enzyme *S*_0.5_ values for ATP were similar to that of the wild type (Table 2). In the presence of 5 mM 3-PGA, the wild type enzyme showed a 6.8-fold decrease in the *S*_0.5_ for ATP (Table 2), which is in agreement with previously reported data for other ADP-Glc PPases from plants [3]. Conversely, the decrease in the *S*_0.5_ for ATP in the mutant enzymes in the presence of 3-PGA was only approximately 2-fold (Table 2). These results agree with those reported for the *E. coli* mutants Q74A and W113A [19], which did not show significant changes in ATP kinetics when Fru-1,6-bisP was included in the reaction media. In the absence of 3-PGA, analysis of the Glc-1P and Mg^2+^ *S*_0.5_ values for the wild type and its Trp mutants showed no significant differences (Table 2). However, in the presence of 5 mM 3-PGA, the wild type enzyme had a 4-fold lower *S*_0.5_ for Glc-1P, whereas the Trp mutants displayed essentially the same kinetics as those observed in absence of activator (Table 2).

**Table 2:** Kinetic parameters for the substrates of wild type potato tuber ADP-Glc PPase and its Trp mutants.

Enzyme	3-PGA	ATP		Glc-1P		Mg^2+^	
		*S*_0.5_ (mM)	*n*_H_	*S*_0.5_ (mM)	*n*_H_	*S*_0.5_ (mM)	*n*_H_
*Stu*S/*Stu*L	none	0.50±0.07	1.4	0.20±0.02	1.3	1.04±0.05	3.7
	5 mM	0.073±0.005	1.2	0.056±0.002	2.0	1.84±0.09	2.4
*Stu*S_W116A_/*Stu*L	none	0.73±0.09	1.1	0.24±0.02	1.3	1.26±0.03	4.6
	5 mM	0.39±0.09	1.2	0.31±0.09	0.9	3.0±0.3	2.4
*Stu*S/*Stu*L_W128A_	none	0.44±0.08	0.8	0.20±0.02	1.4	1.00±0.05	3.7
	5 mM	0.20±0.03	1.2	0.12±0.02	0.9	4.2±0.3	1.8
*Stu*S_W116A_/*Stu*L_W128A_	none	0.87±0.09	1.2	0.25±0.03	1.5	1.40±0.06	3.6
	5 mM	0.33±0.04	1.2	0.19±0.03	1.2	3.3±0.1	2.4

Reactions were performed using *Assay B* in absence of effectors or with the addition of 5 mM 3-PGA, as stated under “Materials and Methods”.

Results of the present work reinforce the proposed mechanism for the allosteric regulation of ADP-Glc PPase, where the mutated residues are important for stabilizing a closed (more active) conformation of the enzyme, thus favoring catalysis. Since the potato tuber enzyme is a heterotetramer, this effect should be observed when the mutations are introduced in the catalytic (S) subunit. However, the mutations in the L subunit also had a minor effect, which suggests the presence of an allosteric signal between the S and L subunits. Indeed, it has been reported that mutations in the regulatory subunit can modulate activity of the enzyme [12], [32], thus confirming that the L subunit is involved in the activation mechanism. The lack of activation displayed by these mutants is not due to the enzyme being in a pre-activated state, as reported for the truncated *E. coli* [33] and chimeric maize/potato [34] ADP-Glc PPases. Essentially, kinetic properties for substrates and activity exhibited by the mutant potato tuber enzymes (Tables 1 and 2) were similar or even lower than the wild type enzyme.

Mutants of the potato tuber ADP-Glc PPase displayed inhibition kinetics for Pi with *I*_0.5_ values between 5.3- and 7.9-fold higher than that obtained for the wild type enzyme (Table 3). The most striking result was observed for the wild type enzyme when Pi curves were performed in the presence of 5 mM 3-PGA: the *I*_0.5_ increased (70-fold), as did the *n*_H_ (3.7-fold). This dramatic effect was not seen for the mutant enzymes when inhibition kinetics were measured in the presence of 3-PGA. The *I*_0.5_ values increased between 1.3- and 5.4-fold, whereas the *n*_H_ values changed from 1.3- to 2.0-fold (Table 3). These results indicate that 3-PGA could not prevent the inhibition elicited by Pi in the mutant enzymes, thus confirming that the activation mechanism was disrupted by the mutations introduced not only in the S but also in the L subunit. The latter highlights the interplay between both subunits for the activation of the wild type enzyme. The residues mutated in this study affected activation and inhibition of the potato tuber ADP-Glc PPase, suggesting that both allosteric effects could share a triggering mechanism that uses the same loop structures. One may favor a more active conformation, and the inhibitor, the opposite. When the structure of the loop is disrupted by mutagenesis, it becomes more insensitive to both signals.

**Table 3:** Kinetic parameters obtained for Pi with the potato tuber ADP-Glc PPase and its mutants.

Enzyme	3-PGA	Pi	
		*I*_0.5_ (mM)	*n*_H_
*Stu*S/*Stu*L	none	0.034±0.003	0.7
	5 mM	2.4±0.3	2.6
*Stu*S_Q75A_/*Stu*L	none	0.18±0.03	0.8
	5 mM	0.48±0.03	1.3
*Stu*S/*Stu*L_Q86A_	none	0.21±0.03	0.9
	5 mM	0.45±0.03	1.2
*Stu*S_W116A_/*Stu*L	none	0.24±0.03	0.8
	5 mM	1.3±0.2	1.6
*Stu*S/*Stu*L_W128A_	none	0.26±0.03	0.9
	5 mM	0.33±0.01	1.5
*Stu*S_W116A_/*Stu*L_W128A_	none	0.27±0.04	0.9
	5 mM	0.71±0.06	1.2

Reactions were performed using *Assay B* in absence or presence of 5 mM 3-PGA, as stated under “Materials and Methods”.

**Figure 4:**
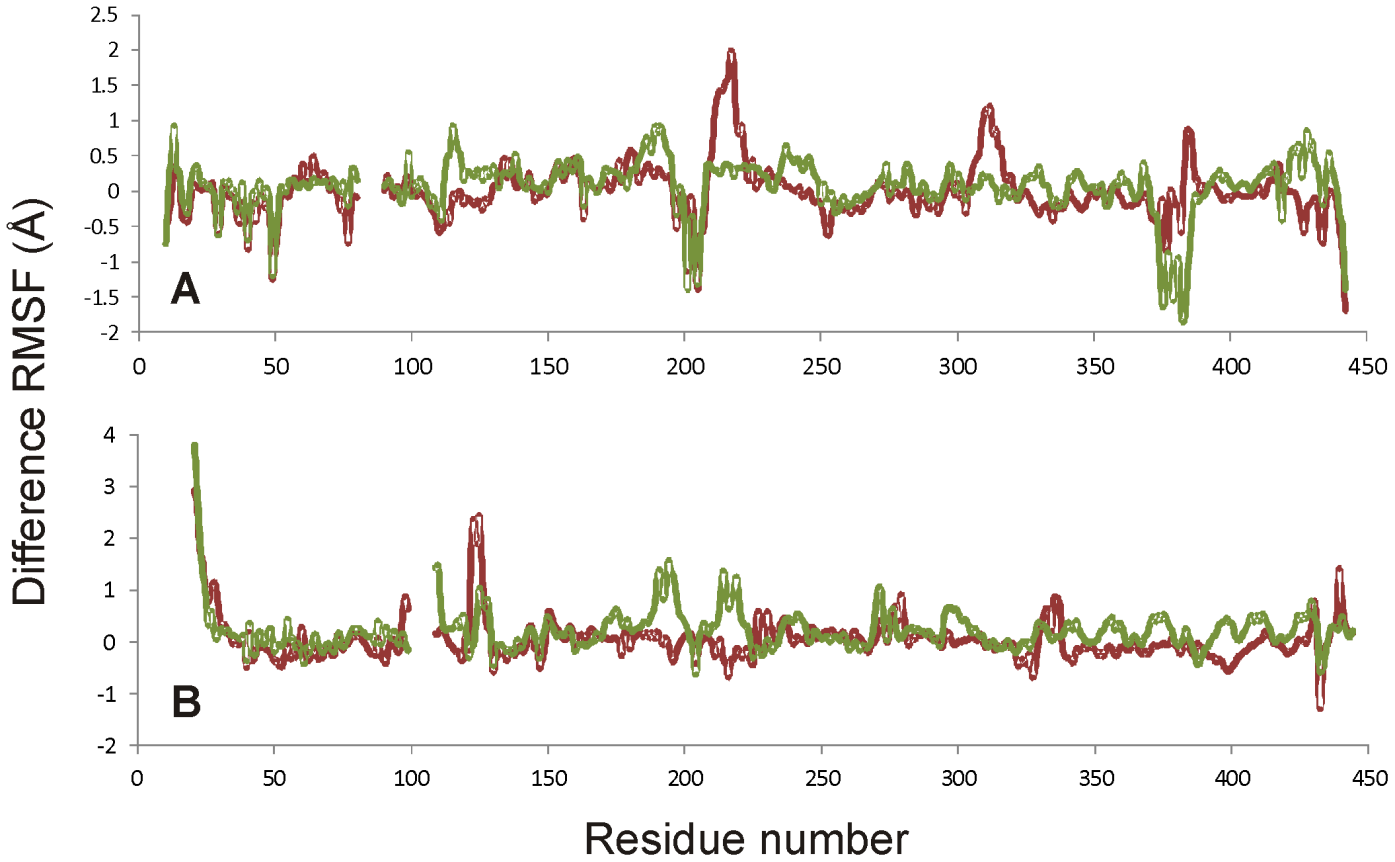
Influence of mutations on difference RMSF values from molecular dynamics simulations. The difference RMSF values were calculated by subtracting the RMSF for the wild type protein from that of the mutant protein. Both panels represent equally spaced sampling of 10 ns simulations. Blank areas at the beginning and near residue 100 represent the sections of the structure that were not present in the original X-ray structure and were not used in calculating the RMSF values. A. Small subunit results; red is *Stu*S_Q75A_ and green is *Stu*S_W116A_. B. Large subunit results; red is *Stu*L_Q86A_ and green is *Stu*L_W128A_.

**Figure 5:**
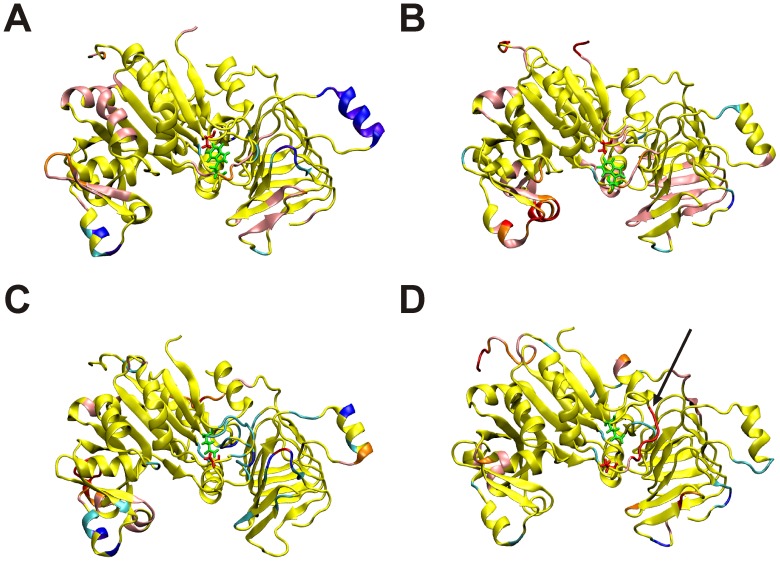
Changes in the molecular dynamics simulations due to mutations in the subunits. The structures are colored according to the difference RMSF values. Colors used are purple, more than 3 standard deviations negative; dark blue, 2 to 3 standard deviations negative; light blue, 1 to 2 standard deviations negative; yellow, +/− one standard deviation; pink, 1 to 2 standard deviations positive; orange, 2 to 3 standard deviations positive; and red, more than 3 standard deviations positive. A. *Stu*S_Q75A_. B. *Stu*L_Q86A_. C. *Stu*S_W116A_. D. *Stu*L_W128A_. The position of the residue mutated to Ala is shown in red, while the non-mutated position is shown in green. The values of the standard deviations were *Stu*S_Q75A_, 0.38; *Stu*L_Q86A_, 0.40; *Stu*S_W116A_, 0.40; and *Stu*L_W128A_, 0.43. Because of the different flexibilities, the Trp-containing loop that was mutated is in different colors. It is shown in red in panel D (arrow).

The architecture of the active site of the potato tuber ADP-Glc PPase crystallized in its inhibited form [13] and of other sugar-nucleotide pyrophosphorylases are almost identical [35]. Also, no significant changes were observed in the catalytic side-chains of the potato tuber ADP-Glc PPase when substrates (ATP or ADP-Glc) were bound [13]. For these reasons, the structural changes responsible for the allosteric control of this enzyme are not obvious. However, there are significant movements of the loops studied in this work, which are near to but do not directly interact with the substrate ATP and are not involved in catalysis. It is not clear how these loops trigger activation in ADP-Glc PPases because positioning of the critical catalytic side-chain residues in the active site does not seem to change. It is possible that the allosteric activation mechanism depends on dynamics, not from specific side-chain orientations, but from the protein’s conformational flexibility (backbone and side chain mobility). That is, the residues of the activated enzyme form could have specific changes of mobility (or rigidity) that is different from the non-activated form. Based upon this, we hypothesize that the flexibilities of the loops in question may be important for activation of this enzyme. In support of our hypothesis, we have performed computational experiments (molecular dynamics simulations) and have observed significant changes in the flexibilities of these loops in both the S and L subunits due to the mutations studied here. Figure 4 shows the difference between the RMSFs of the mutant and native subunits. All four mutations (*Stu*S_Q75A_, *Stu*L_Q86A_, *Stu*S_W116A_, and *Stu*L_W128A_) resulted in changes in the flexibilities of the subunits. Some of these changes, particularly for the *Stu*S_W116A_ chain (Figure 4A), were decreases in flexibility, while most show specific areas of increasing flexibility. The RMSF changes are mapped onto the three-dimensional structure in Figure 5. In this diagram the regions in yellow show flexibilities that are very similar in the wild-type and mutant proteins, whereas the other colors show regions of changed flexibility. *Stu*L_Q86A_, which is the mutation that shows little effect on the 3-PGA saturation curve (Figure 3), has the least flexibility change (Figure 5B) for the Trp-containing loop (Figure 1B). Both increases and decreases in the flexibility of this loop compared to the wild-type structure (Figure 5A, C, and D) correlate with changes in the 3-PGA saturation curves (Figure 3). Thus, it may be that the allosteric relation of this enzyme depends on having the correct degree of dynamics in this Trp-loop (Fig. 1B).

One of the first examples of allosterism in plants was the regulation of the spinach leaf ADP-Glc PPase by 3-PGA and Pi [36]–[38]. The regulatory properties of that enzyme were found almost simultaneously with the publication of the Monod-Wyman-Changeux model for allosterism [39]. Despite these early findings, it has not been possible to describe a structural mechanism for the regulation of this enzyme, but important progress has been made since X-ray structures were solved [13], [17]–[19]. Our current understanding of the allosteric activation of the ADP-Glc PPase from bacteria [19] and from plants (this work) shares the view that proteins are in constant motion [40] and that dynamics are critical for signaling and allosterism [41], [42].

## Supporting Information

Table S1 **Oligonucleotides used for introducing mutations into the sequences coding for the** ***Anabaena*** **sp. PCC 7120 and potato tuber (** ***Stu*** **S and** ***Stu*** **L subunits) ADP-Glc PPases.**
(DOCX)
