# Supplementary material for: Unraveling the Activation Mechanism of the Potato Tuber ADP-Glucose Pyrophosphorylase
Source: PLoS One. 2013 Jun 24;8(6):e66824. doi: 10.1371/journal.pone.0066824 (PMC3691274; doi:10.1371/journal.pone.0066824)
Supplement: Table S1 — Oligonucleotides used for introducing mutations into the sequences coding for the Anabaena sp. PCC 7120 and potato tuber ( Stu S and Stu L subunits) ADP-Glc PPases. (DOCX) [file pone.0066824.s001.docx]

**Table S1.** Oligonucleotides used for introducing mutations into the sequences coding for the *Anabaena*sp. PCC 7120 and potato tuber (*Stu*S and *Stu*L subunits) ADP-Glc PPases.

| Oligonucleotide | Sequence | Mutation | Restriction  site |
| --- | --- | --- | --- |
| *Ana*-Q58A-fo | ATC TAC GTA TTA ACA GCA TTT AAC TCA GCT TCT | Q58A | none |
| *Ana*-Q58A-re | AGA AGC TGA GTT AAA TGC TGT TAA TAC GTA GAT | Q58A | none |
| *Ana*-W96A-fo | CCA GAG AAC CCT AAC GCG TTC CAA GGT ACA GCC | W96A | none |
| *Ana*-W96A-re | GGC TGT ACC TTG GAA CGC GTT AGG GTT CTC TGG | W96A | none |
|  |  |  |  |
| *Stu*S-fo | CAT ATG GCT GTT TCT GAT TCG CAG A | none | *Nde*I |
| *Stu*S-re | GAG CTC AGA TGA TGA TTC CAC TTG G | none | *Sac*I |
| *Stu*S-*Nde*I-fo | ACC TTT CAC GAG CGT ATG CTA GCA A | *Nde*I deletion | none |
| *Stu*S-*Nde*I-re | TTG CTA GCA TAC GCT CGT GAA AGG T | *Nde*I deletion | none |
| *Stu*S-Q75A-fo | GTT CTC ACA GCA TTC AAC TCT GCC T | Q75A | none |
| *Stu*S-Q75A-re | AGG CAG AGT TGA ATG CTG TGA GAA C | Q75A | none |
| *Stu*S-W116A-fo | GAG AAC CCC GAT GCG TTC CAG GGC A | W116A | none |
| *Stu*S-W116A-re | TGC CCT GGA ACG CAT CGG GGT TCT C | W116A | none |
|  |  |  |  |
| *Stu*L-fo | CAT ATG GCT TAC TCT GTG ATC ACT AC | none | *Nde*I |
| *Stu*L-re | GAG CTC GAA TTC TCA TGT TTG ACA G | none | *Sac*I |
| *Stu*L-*Nde*I-fo | GAT GGA ACA GTC ATC TGA ACT AGG G | *Nde*I deletion | none |
| *Stu*L-*Nde*I-re | CCC TAG TTC AGA TGA CTG TTC CAT C | *Nde*I deletion | none |
| *Stu*L-Q86A-fo | TTG TGC TGA CAG CGT ACA ATT CTG C | Q86A | none |
| *Stu*L-Q86A-re | GCA GAA TTG TAC GCT GTC AGC ACA A | Q86A | none |
| *Stu*L-W128A-fo | AGC AGG AAA AAA AGC GTT TCA AGG A | W128A | none |
| *Stu*L-W128A-re | TCC TTG AAA CGC TTT TTT TCC TGC T | W128A | none |
